# Vertical Profiles of Soil Water Content as Influenced by Environmental Factors in a Small Catchment on the Hilly-Gully Loess Plateau

**DOI:** 10.1371/journal.pone.0109546

**Published:** 2014-10-14

**Authors:** Bing Wang, Fenxiang Wen, Jiangtao Wu, Xiaojun Wang, Yani Hu

**Affiliations:** 1 College of Environmental Science and Resources, Shanxi University, Taiyuan, China; 2 Library, Hebei University of Science and Technology, Shijiazhuang, China; NERC Centre for Ecology & Hydrology, United Kingdom

## Abstract

Characterization of soil water content (SWC) profiles at catchment scale has profound implications for understanding hydrological processes of the terrestrial water cycle, thereby contributing to sustainable water management and ecological restoration in arid and semi-arid regions. This study described the vertical profiles of SWC at the small catchment scale on the hilly and gully Loess Plateau in Northeast China, and evaluated the influences of selected environmental factors (land-use type, topography and landform) on average SWC within 300 cm depth. Soils were sampled from 101 points across a small catchment before and after the rainy season. Cluster analysis showed that soil profiles with high-level SWC in a stable trend (from top to bottom) were most commonly present in the catchment, especially in the gully related to terrace. Woodland soil profiles had low-level SWC with vertical variations in a descending or stable trend. Most abandoned farmland and grassland soil profiles had medium-level SWC with vertical variations in varying trends. No soil profiles had low-level SWC with vertical variations in an ascending trend. Multi-regression analysis showed that average SWC was significantly affected by land-use type in different soil layers (0–20, 20–160, and 160–300 cm), generally in descending order of terrace, abandoned farmland, grassland, and woodland. There was a significant negative correlation between average SWC and gradient along the whole profile (*P*<0.05). Landform significantly affected SWC in the surface soil layer (0–20 cm) before the rainy season but throughout the whole profile after the rainy season, with lower levels on the ridge than in the gully. Altitude only strongly affected SWC after the rainy season. The results indicated that land-use type, gradient, landform, and altitude should be considered in spatial SWC estimation and sustainable water management in these small catchments on the Loess Plateau as well as in other complex terrains with similar settings.

## Introduction

Soil water content (SWC) is a critical factor for plant growth and a determinant of plant distribution in arid and semiarid areas such as China's Loess Plateau [1], [2]. Vertical distribution of SWC can greatly affect soil water movement [3], thereby greatly affecting the biomass production and water use efficiency of plants (e.g., switchgrass) under water stress [4]. Plant-available water stored in the soil profile has a buffering capacity, which, in deep layers, prolongs or alleviates the effects of seasonal or inter-annual drought on plant growth and soil water flux to the atmosphere [5]–[7]. Research has provided strong evidence that deep soil water depletion plays a key role in sustainable agriculture, ecological restoration, and terrestrial water cycling on the Loess Plateau [8]–[10]. However, measurement of SWC profiles has been frequently conducted at different spatial scales. The results thus need to be converted before comparison analysis or practical uses. The SWC profile in small catchment is considered to be at a moderate scale for data exchanging. In particular, small catchment is thought to be the basic unit for integrated soil and water loss management in complicated terrain of the Loess Plateau [11], [12]. Characterization of SWC profiles and evaluation of relevant influencing factors at the small catchment scale have implications for hydrological modeling of soil water dynamics and sustainable management of soil water resources in similar areas.

Classical statistics is frequently used to analyze the variability of SWC profiles at the small catchment scale, which involves the estimation of descriptive parameters such as average (mean), variance, standard deviation (STD), and coefficient of variation (CV). Average SWC at individual soil depth intervals or across the whole soil profile is extensively determined. The CV of SWC is also routinely calculated as the temporal variable in a certain period of time or the spatial variable across a specific area. The SWC profile can be divided into distinct intervals by considering its average and CV which exhibit complex spatial-temporal relationships in several plots or watersheds [13]–[16]. Additionally, ranking method, clustering method, and semivariogram model have been applied for the division of SWC profile [3], [17]–[20]. However, the above-mentioned methods cannot clearly reflect the variation trend in SWC profiles. Thus, great effort has been made to describe the vertical profiles of SWC through comparing variation curves or variation ranges, in small watersheds related to different land-use types, vegetation species, and/or terrain factors [3], [17], [18], [20]–[22]. If a massive sample size is involved, however, it becomes difficult to distinguish the vertical profiles and major influencing factors of SWC by direct comparisons.

In recent decades, a great number of studies have been conducted on the spatiotemporal variability of SWC and related influencing factors worldwide. Canton [23] pointed out that wasteland-scale spatial variability of SWC is mainly controlled by surface cover and soil properties in a semi-arid region of Spain, where surface cover counteracts the influence of terrain factors (including gradient, aspect, topographic wetness index, and distance from the river) on SWC distribution. Burnt [24] reported a topographic index which can simulate changes in high-level SWC in a humid climate zone of Devin County, UK. O'loughlin [25] estimated the spatial pattern of SWC distribution in a small catchment using humidity index model based on digital terrain dataset. Hawley [15] discovered that topography is the major factor responsible for the spatial distribution of SWC in an agricultural region, where resultant SWC variation is diminished by vegetation in a moist climate zone in Chickasha, USA. In arid and semi-arid areas, catchment-scale distribution of SWC is strongly affected by land-use/vegetation and topographic indices, e.g., land-use type, soil organic matter content, tillage, soil physical properties, gradient, and aspects [6], [17]–[19], [21], [26].

Many researchers have focused on the quantification of environmental parameters such as topographic factor, vegetation type, soil texture, and land-use type, in attempt to evaluate their impacts on the variability of SWC. At the small catchment scale, little information is available on the major factors affecting vertical profiles of SWC in cinnamon soil (Haplic Lixisols, FAO) zone on the hilly and gully Loess Plateau [3], [19]. As a regional water reservoir experiencing depletion, the plateau region requires measurements and characterization of deep SWC profiles for the thick soil layer. However, soil sampling for SWC profile analysis at the catchment scale has been commonly conducted at <200 cm depth [18]–[20], [23]. Wang et al. [21] exceptionally examined SWC along the 0–21 m soil profile on the Loess Plateau, but the reliability of their tests might be affected by a small sample size (11 sites). Deep soil sampling at a larger number of sites and statistical analysis of parameters involving soil depth information will contribute to better understanding of the vertical profiles and influencing factors of SWC.

In the present study, we characterized the vertical profiles of SWC in a small catchment on the Loess Plateau by cluster analysis of two descriptive parameters (mean and regression gradient). Sampling was carried out in the 0–300 cm profile at 101 points throughout the catchment before and after the rainy season, to meet the demand for deep depth, spatial representativeness and temporal comparability. The influences of selected environmental factors (land-use type, topographic factors, and landform) on average SWC were examined by multi-linear regression [6], [26]–[32]. The results were discussed in order to provide new insights to the vertical profiles and influencing factors of SWC on the Loess Plateau, further providing reference data for sustainable management of water resource in small catchment areas in the semi-arid region with complex terrain.

## Material and Methods

### 1 Site description

**Figure 1:**
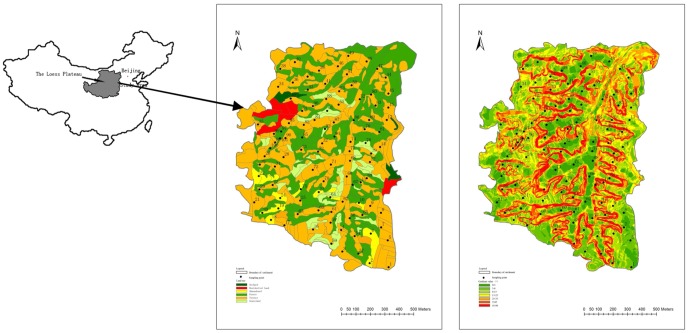
Location of Sanyanjing catchment and distribution of 101 sampling points in the Sanyanjing catchment.

This study was conducted in Sanyanjing catchment (112°2′13″, 37°46′23″), which is located on the east margin of the Loess Plateau in Shouyang county, mid-east Shanxi province, China (Figure 1). The catchment has a total area of 1.32 km^2^ and the elevation ranges from 1001 to 1160 m. It is a hilly and gully area with mostly deep gully erosion slopes. The landform consists of ridge and gully.

**Figure 2:**
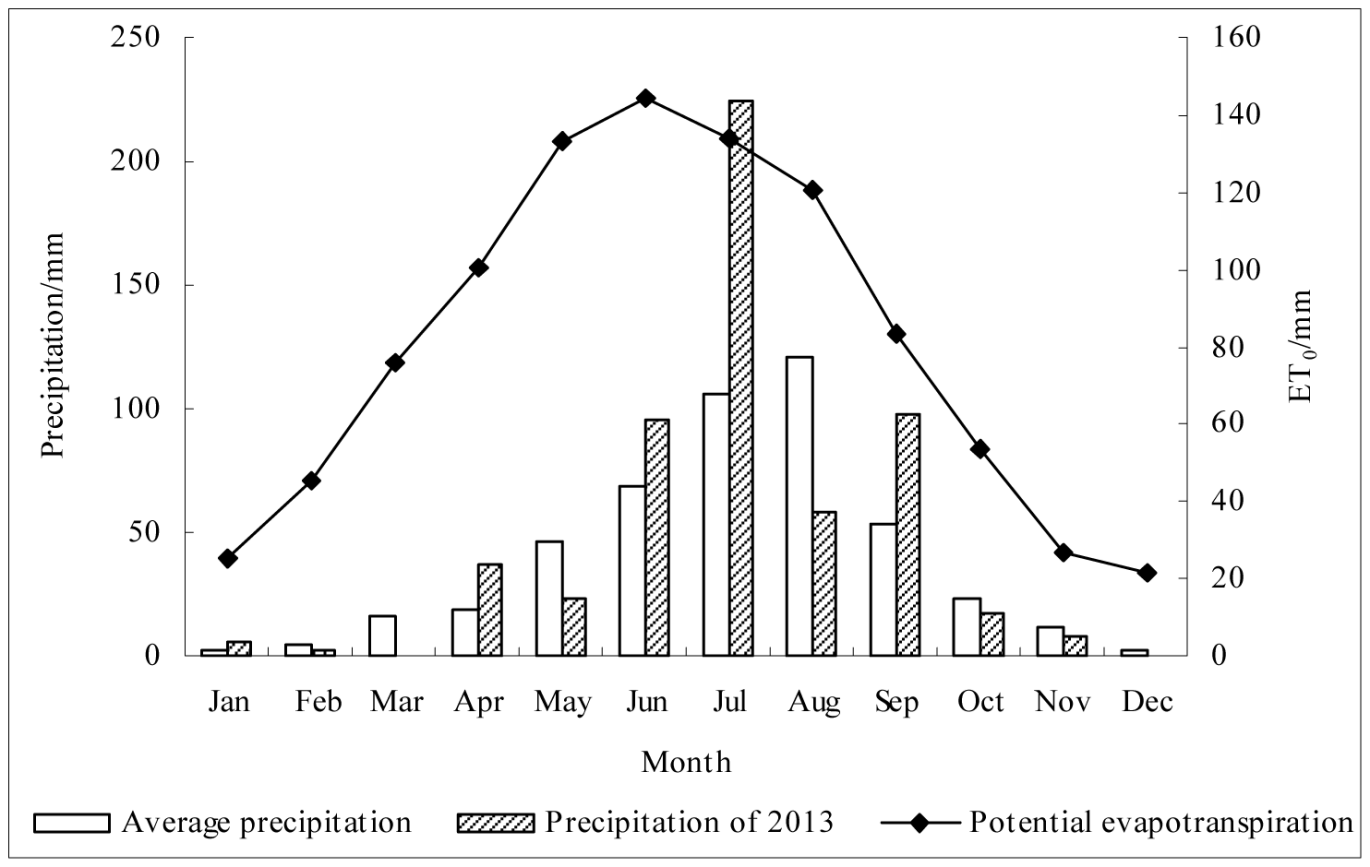
Average annual precipitation and potential evapotranspiration of 1967–1999 and precipitation in 2013 in the Sanyanjing catchment, Shanxi province, China.

The catchment area has a semi-arid continental climate (Cwa by Koppen Climate Classification) with an average annual precipitation of 474.2 mm (1967–1999). Snow in the winter accounts for ∼8% and rainfall in July to September for ∼73% of annual precipitation. Monthly average precipitation, potential evapotranspiration and precipitation in 2013 are shown in Figure 2. Annual mean temperature in this area is 8.1°C, with a maximum of 34.7°C and a minimum −20.6°C. The soil type is cinnamon soil (Haplic lixisols, FAO), which consists of 54–62% silt and 10.95–30.15% sand with the bulk density of 1.3–1.4 g/cm^3^. Soil texture was measured using a particle size analyzer (SEDIMAT 4–12, UGT, Germany). Soil bulk density was determined through sampling with cutting rings (inner diameter 5.0 cm, volume 100 cm^3^) and drying in an oven (105°C, 24 h). The profile of soil texture related to different landforms is listed in Table 1. Maximum soil depth is mostly down to 300 cm on the ridge and bare rock could be rarely seen only on the northern margin of the gully area.

**Table 1:** Soil texture in vertical profiles related to different landforms in the Sanyanjing catchment in Shanxi province, China.

Point description	Soil depth (cm)	Sand (%)		Silt (%)		Clay (%)
		>0.05 mm (%)	0.05–0.02 mm (%)	0.02–0.0063 mm (%)	0.0063–0.002 mm (%)	<0.002 mm (%)
	0–20	26.05	30.40	20.60	7.00	15.95
	20–40	21.35	34.80	20.80	6.90	16.15
	40–60	30.25	25.90	21.60	4.50	17.75
	60–80	28.25	28.80	21.70	2.80	18.45
	80–100	18.45	38.00	20.80	6.70	16.05
	100–120	26.45	30.10	20.40	5.20	17.85
Terrace at	120–140	26.95	27.90	22.00	4.40	18.75
ridge	140–160	16.35	32.60	24.20	8.10	18.75
(Point 33)	160–180	27.75	22.40	22.70	7.90	19.25
	180–200	36.55	17.80	20.60	8.00	17.05
	200–220	18.45	29.00	24.30	9.00	19.25
	220–240	20.45	28.50	23.20	8.60	19.25
	240–260	22.05	27.30	23.90	8.80	17.95
	260–280	31.35	20.30	22.20	9.40	16.75
	280–300	24.65	29.80	20.70	5.90	18.95
	0–20	30.15	20.00	23.20	6.40	20.25
	20–40	27.75	21.80	21.90	6.10	22.45
	40–60	25.95	26.80	20.50	4.50	22.25
	60–80	23.85	27.09	21.20	1.20	26.65
	80–100	0.95	31.00	20.10	20.10	27.85
	100–120	10.95	23.40	18.40	18.40	28.85
Terrace at	120–140	7.45	27.30	19.20	19.20	26.85
ridge	140–160	17.45	19.50	18.90	18.90	25.25
(Point 39)	160–180	10.45	25.80	18.20	18.20	27.35
	180–200	15.75	24.70	17.00	17.00	25.55
	200–220	13.35	21.40	18.60	18.60	28.05
	220–240	20.85	8.80	22.00	22.00	26.35
	240–260	20.25	12.40	20.00	20.00	27.35
	260–280	12.15	23.70	19.10	19.10	25.95
	280–300	24.95	29.00	20.50	0.50	25.05

The distribution of land-use types across the catchment is shown in Figure 1. Terrace is the dominant land-use type, accounting for about 60% of the study area. Few terraces had been abandoned for natural restoration of vegetation because of the Grain for Green project since 2000. Grassland is mainly covered with herbs and semi-shrubs, which had never been reclaimed for several decades. About 80% of the woodland is covered with semi-shrubs at steep slopes unsuitable for sampling.

### 2 Soil sampling

#### Ethics statement

Sampling activities at the farmland were allowed by the owners. No specific permissions were required at other locations because they were not privately-owned or protected in any way and the field activities did not involve any endangered or protected species.

A total of 101 sampling points were designed in a 150 m×150 m grid throughout the catchment area by considering major land-use types, including terrace (83), abandoned farmland (9), grassland (3), and woodland (6). Soil sampling was carried out during two periods in 2013, from April 29 to May 4 (before the rainy season) and from October 28 to November 1 (after the rainy season). No precipitation occurred during the two sampling periods or a week before sampling. Each sample was taken at 20 cm intervals along the 0–300 cm soil profile using an auger (inner diameter 5.0 cm). The samples were kept in capped aluminum boxes for transportation. Measurement of SWC was conducted using an oven-drying method (105°C, 24 h). At the majority of the sampling points, soils were collected along a vertical profile over 300 cm. A few exceptions were in the north of the gully at the lowest altitude where weathered rock was occasionally encountered. Background information of the 101 sampling points is summarized in Table 2.

**Table 2:** Background information of 101 soil sampling points in the Sanyanjing catchment study area in Shanxi province, China.

Land-use type	Vegetation	Landform type	Soil profile/cm	Sampling points
**Terrace (n = 83)**	Maize	Ridge	300	1, 3–5, 7–9, 12–18, 21–22, 25–26, 28, 32–35, 37, 51, 53, 91, 95, 96, 98
			260	99
		Gully	300	29, 30, 38–46, 48–50, 54–60, 62, 63, 65, 66, 68–74, 77–84, 87–90
			280	47
			260	31, 61
			220	52
			160	86
	Millet	Ridge	300	11
	Maize +five-year-walnut	Gully	300	27
				
**Abandoned farmland**	Subshrubs + herbs	Ridge	300	6, 10, 23
**(n = 9)**			240	2
	Subshrubs + herbs + few ulmus pumila	Gully	220	19
	*Robinia peseudoacacia* + subshrubs + herbs	Ridge	300	24
	Robinia peseudoacacia+ subshrubs + herbs	Gully	300	64
	Herbs + few almond-apricot	Gully	300	20
	Poplar + subshrubs +herbs	Gully	300	75
**Grassland**	Subshrubs +herbs	Ridge	300	92, 93
**(n = 3)**		Gully	300	76
**Woodland**	Poplar	Gully	300	97
**(n = 6)**			280	85
	Poplar + herbs	Gully	140	101
	Poplar + subshrubs	Gully	280	100
	Poplar + subshrubs + herbs	Gully	300	67, 94

### 3 Data analysis

To identify the variability of SWC profiles at the catchment scale, descriptive parameters were calculated for each profile. Further, we calculated the linear regression coefficient (K value) between SWC and soil depth to represent the variation trend of SWC vertical profiles and the mean value to describe the average level of SWC along the 0–300 cm profile.

The SWC profiles were classified using a combined cluster analysis of the K and mean values. Cluster analysis is the process of grouping a set to data objects into multiple groups (or clusters), so that objects within a cluster share high similarity but are dissimilar to those in other clusters. In this approach, dissimilarities and similarities are assessed based on the attribute values describing the objects and often involve distance measures. Cluster analysis is a statistical classification method for discovering whether the individuals of a population gall into different groups by making quantitative comparisons of multiple characteristics [34]. Here a combined cluster analysis was conducted in three steps: 1) cluster of the mean to three groups, which present the average level of SWC along the vertical soil profile, 2) cluster of K to three groups, which reflect the variation trend of SWC profiles (top to bottom), and 3) combination of the two sets of groups into nine new groups using the between-groups linkage method with squared Euclidean distance criteria [34].

For group comparisons, SWC profiles (0–300 cm) of the same group were averaged and re-plotted. The average curves of SWC were compared between groups to identify the major factors influencing SWC in individual soil layers (0–20, 20–160, and 160–300 cm). On the basis of cluster analysis, the influences of land-use type, topography and landform on average SWC in individual soil layers were examined by multi-regression analysis. The independent variables were land-use type, landform type, Sin(gradient), Sin(aspect), flow accumulation (calculated cell numbers to a grid cell from surrounding cells with the ArcGIS hydrology analysis module), and elevation. The former two factors were categorical variables converted into dummy variables before introduced into the regression analysis; and the latter four factors were continuous variables produced using digital elevation model at 1-m resolution.

SWC data were statistically analyzed in SPSS13.0 (SPSS Inc., Chicago, IL, USA), and topographic features were analyzed in ArcGIS 10.0 (ESRI, Redlands, CA, USA). SWC profiles were drawn in Microsoft Excel 2010 (Microsoft Corp., Redmond, WA, USA) and then clustered in SPSS 13.0 by considering descriptive parameters (maximum, minimum, mean, CV, STD, and K). Multi-regression analysis was performed in SPSS 13.0, with a *P-*value less than 0.05 considered statistically significant.

## Results

### 1 Vertical profiles and descriptive parameters of SWC

**Figure 3:**
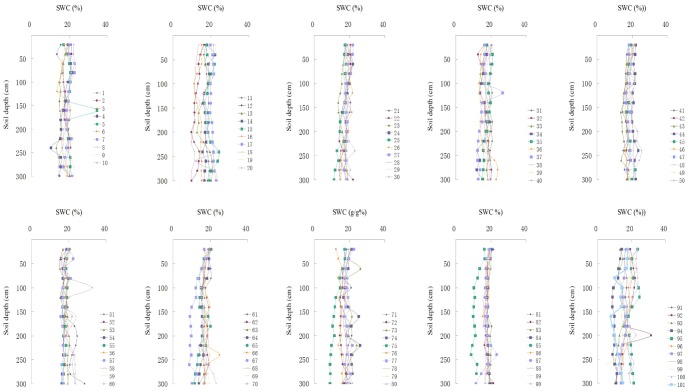
Vertical profiles of soil water at 101 sampling points in the Sanyanjing catchment.

The vertical profiles (0–300 cm) of SWC at 101 sampling points before the rainy season were drawn (Figure 3). These SWC profiles showed dynamic variations across the catchment study area, with substantial differences in the soil layers below 100 cm. At a few sampling points, there were obvious soil water depletion (e.g., 67, 75, 85, and 94) and an increasing trend (top to bottom) of SWC (e.g., 12 and 36). High degrees of soil desiccation were rarely detected in the lower soil layers, and low SWC was mainly found in the lower soil layers of woodland.

**Figure 4:**
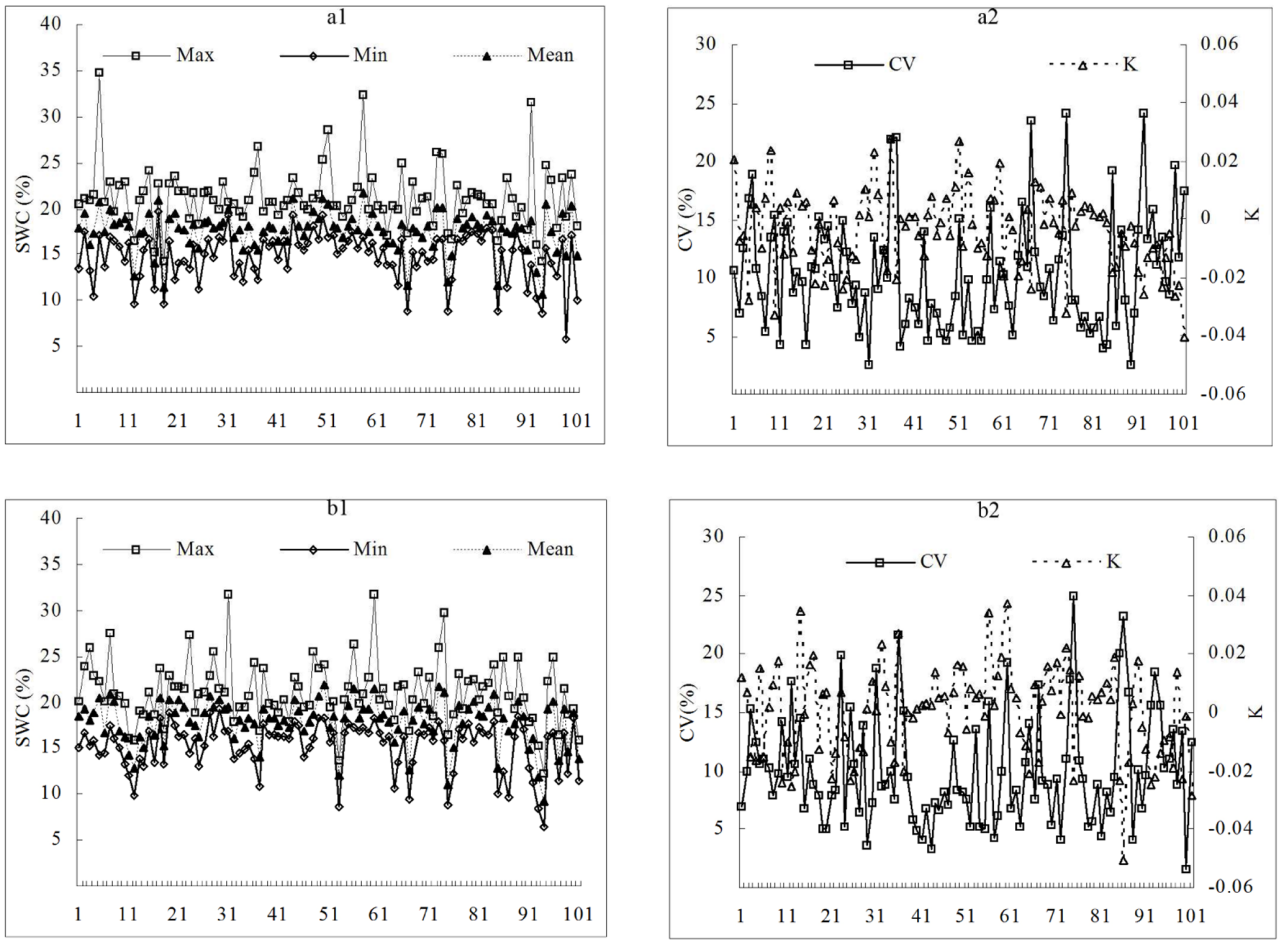
Statistical parameters of soil water content at 101 sampling points across the Sanyanjing catchment. (a. before the rainy season; and b. after the rainy season.)

Descriptive parameters such as maximum, minimum, mean, and CV, STD are commonly used to reveal the spatial-temporal variability of SWC. However, these parameters cannot reflect the variation trend of SWC vertical profiles. To this end, the K value of SWC to profile depth was introduced for quantification of variation trend of SWC vertical profiles (Figure 4). Results showed that before the rainy season, SWC substantially varied between 5.87% and 34.72%, whereas the mean, STD, CV, and K values respectively ranged from 10.57% to 21.76%, 0.47 to 4.53, 3% to 24%, and −0.0405 to 0.0274 along the vertical soil profile (0–300 cm). The ranges of the parameters after the rainy season were generally similar with those before the rainy season.

According to the division criteria of Nielson [24], CV in the range of 10–100% indicates moderate variability. Thus, the vertical variability of SWC at all sampling points in Sanyanjing catchment (Figure 4) can be classified to the medium degree. K is the linear regression coefficient between SWC and soil depth. A positive value of K indicates that SWC increases with increasing soil depth. Inversely, a negative value of K indicates that SWC decreases with increasing soil depth. The positive and negative K values of SWC data (Figure 4) are indicative of different variation trends of SWC vertical profiles in the catchment.

### 2 Clustering of SWC profiles

**Figure 5:**
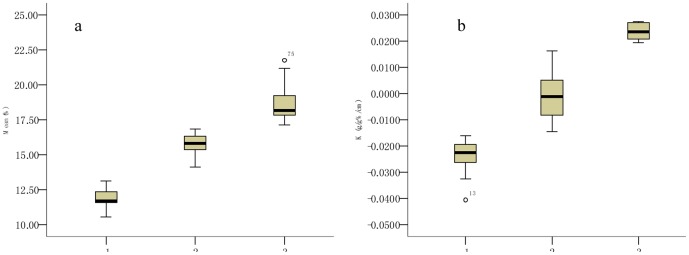
Grouping of 101 vertical soil water profiles in the Sanyanjing catchment before the rainy season by cluster analysis of the mean value (a) and regression gradient (K, b).

The vertical profile of SWC across the catchment can be described more clearly using cluster analysis. The 101 SWC profiles before the rainy season were classified into the first three groups by considering the mean value of SWC (Figure 5a), and the second three groups by considering the K value of SWC to soil depth (Figure 5b). The mean value of SWC ranged from 10.57% to 13.13% (low level), 14.15% to 16.86% (medium level), and 17.13% to 21.76% (high level) in the first three groups, whereas the K value of SWC to soil depth ranged from −0.0405 to 0.0106 (decreasing trend), 0.0144 to 0.0163 (stable trend), and 0.0194 to 0.0274 (increasing trend) in the second three groups. By combining the two cluster series, we obtained nine groups of SWC profiles (Table 3).

**Table 3:** Combined grouping of 101 vertical profiles of soil water content (0–300 cm) in Sanyanjing catchment by cluster analysis of the mean value and regression gradient.

Cluster by mean	Cluster by K	Combined grouping		Quantity of points			Point Nos.	
			Before the rainy season		After the rainy season	Before the rainy season		After the rainy season
1	1	1	3		0	65,67, 85		-
	2	2	4		1	12,18,93,94		94
	3	3	0		0	-		-
2	1	4	6		15	25,37,64,91,99,101		11,12,18,37,53,64,67,75,76,85,91,93,97,99,101
	2	5	13		0	3,16,23,34,41,43,56,63,65,70,72,76,97		3,16,23,34,41, 43,56,63,65,70,72,76,97
	3	6	2		1	32,36		14
3	1	7	11		1	4,10,19,21,26,35,61,86,92,96,100		86
	2	8	58		80	2,5–8,11,13–15,17, 20,22,24,27–31,33, 38–40,42,44–50,52, 53,55,56–59,62,66, 68,69,71,73,74, 77–84,87–90,95,98		2–10,13,15,16, 17,19–36,38–52,54,55,57–59, 61–63,65,66,68,69–74,77–84, 87–90,92,95,96,98,100
	3	9	4		3	1,9,51,60		1, 51,60

Before the rainy season, vertical profiles of SWC in groups 1–3 featured low-level SWC (Table 3). In group 1, SWC decreased along the vertical profile (0–300 cm) in woodland (2) and abandoned farmland (1) located in the gully area. In group 2, SWC remained stable along the vertical profile in terrace (2) and grassland (1) located on the ridge as well as woodland (1) located in the gully (Table 4). No sampling points were classified into group 3 with increasing SWC along the vertical profile.

**Table 4:** Grouping richness of 101 vertical profiles of SWC (0–300 cm) in relation to different land-use types in Sanyanjing catchment before the rainy season.

Land-use type	Grouping								
	1	2	3	4	5	6	7	8	9
Terrace	0	2	0	4	10	2	7	54	4
Abandoned	1	0	0	1	1	0	2	0	0
Grassland	0	1	0	0	1	0	1	0	0
Woodland	2	1	0	1	1	0	1	0	0

Vertical profiles of SWC in groups 4–6 featured medium-level SWC (Table 3). In group 4, SWC decreased along the vertical profile on the ridge related to terrace (4) as well as in the gully related to abandoned farmland (1) and woodland (1) located in the gully. In group 5, SWC remained stable along the vertical profile in terrace (7) mostly located in the gully, few terrace (3) and abandoned farmland (1) located on the ridge, and grassland (1) and woodland (1) located in the gully. In group 6, SWC increased along the vertical profile in terrace (2) located on the ridge (Table 4).

Vertical profiles of SWC in groups 7–9 featured high-level SWC (Table 3). In group 7, SWC decreased along the vertical profile in terrace (5), grassland (1), and abandoned farmland (1) located on the ridge, and terrace (2), abandoned farmland (1) and woodland (1) located in the gully. In group 8, SWC remained stable along the vertical profile at up to 58 of 101 sampling points, far more than other groups. Most sampling points of group 2 were located in the gully related to terrace (41), and few were on the ridge related to terrace (14) and abandoned farmland (3). In group 9, SWC increased along the vertical profile in terrace located on the ridge (3) and in the gully (1) (Table 4).

Similar grouping of SWC profiles was obtained with data collected after the rainy season. Overall, soil profiles of group 8 with high-level SWC in a stable trend were most commonly present in the catchment, more after the rainy season than before the rainy season. Group 3 of SWC profiles with low level and increasing trend was absent in the study area.

### 3 The relationships between average SWC and selected environmental factors

**Figure 6:**
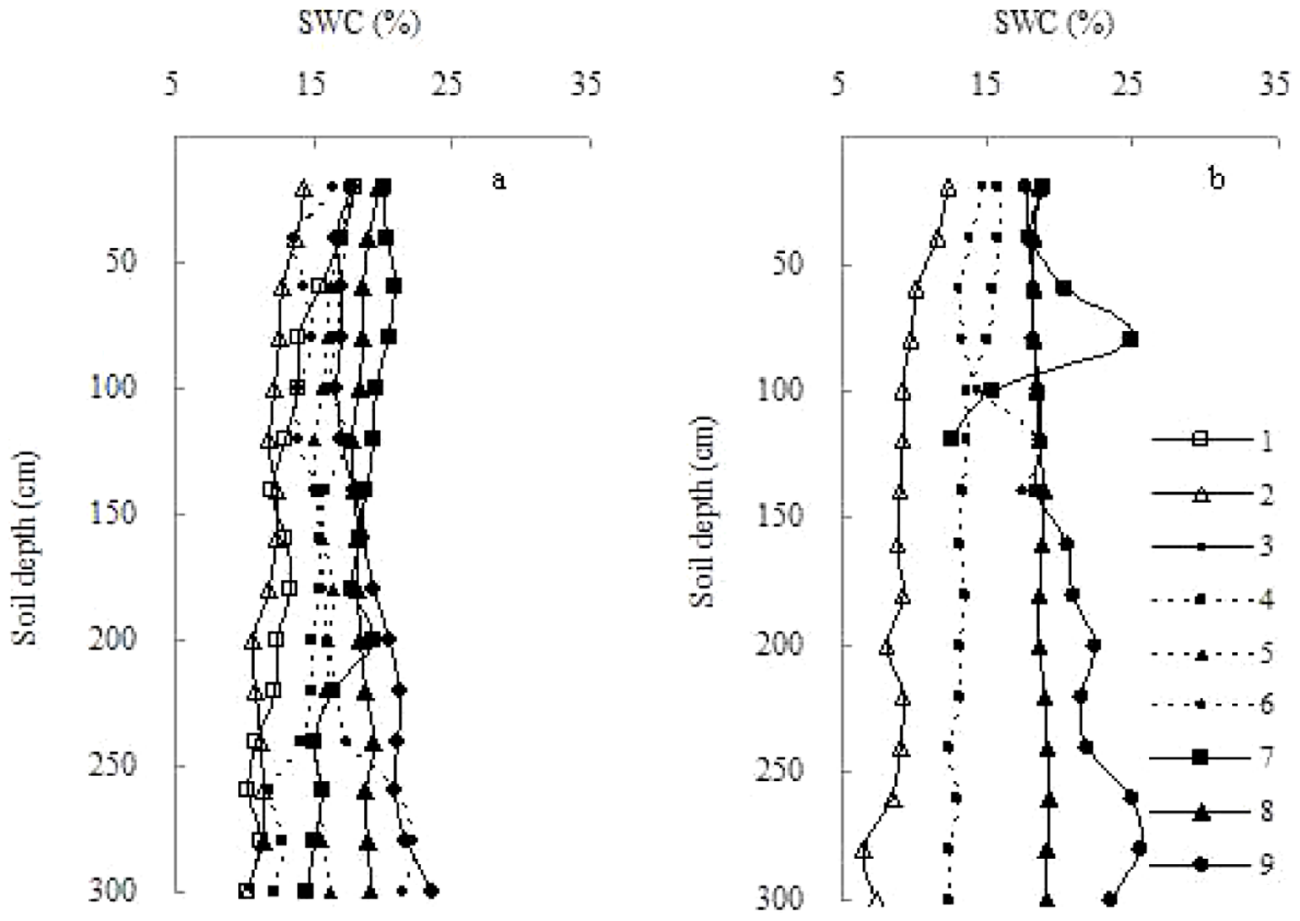
Vertical soil water profiles in relation to different groups in the Sanyanjing catchment study area before and after the rainy season (a. before the rainy season; and b. after the rainy season).

According to cluster analysis, there were nine combinations of SWC profiles in terms of average level and variation trend. We averaged SWC profiles of the same group and plotted the average curves (Figure 6), to examine differences of SWC profiles among various types. From Figure 4, we divided the whole soil profile (0–30 cm) into three layers (0–20, 20–160, and 160–300 cm) for multiple linear regression analysis. The results showed that selected environmental factors had significant linear correlations with average SWC at individual layers of 0–20 cm (*P*<0.001, *R* ^2^ = 0.30; *P*<0.001, *R* ^2^ = 0.37), 20–160 cm (*P* = 0.01, *R* ^2^ = 0.19; *P*<0.001, *R* ^2^ = 0.39), and 160–300 cm (*P*<0.001, *R* ^2^ = 0.32; *P*<0.001, *R* ^2^ = 0.43; Table 5).

**Table 5:** Multi-linear regression analysis of soil water content and selected environmental factors in three layers (0–20, 20–160, and 160–300 cm) of the vertical soil profile in the Sanyanjing catchment study area.

				Before the	rainy season				
Model		Y_1_			Y_2_			Y_3_	
	Unstandardized coefficients B	Standardized coefficients (Beta)	Sig.	Unstandardized coefficients B	Standardized coefficients (Beta)	Sig.	Unstandardized coefficients B	Standardized coefficients (Beta)	Sig.
Constant	14.714		0.066	6.031		0.498	18.514		0.069
X_1_	−2.743	−0.23	0.013	−2.751	−0.22	0.026	−4.471	−0.296	0.001
X_2_	−0.112	−0.037	0.68	0.042	0.013	0.889	−0.261	−0.068	0.449
X_3_	4.86E–06	0.025	0.783	−7.80E–06	−0.038	0.694	1.20E–05	−0.05	0.578
X_4_	0.005	0.081	0.507	0.011	0.177	0.175	0	0.005	0.97
D_51_	1.09	0.146	0.109	0.019	0.002	0.98	−0.788	−0.084	0.354
D_52_	−3.472	−0.278	0.003	−0.845	−0.064	0.503	−2.553	−0.162	0.073
D_53_	−1.715	−0.191	0.047	−3.216	−0.342	0.001	−4.745	−0.384	0
D_61_	−1.512	−0.348	0.005	−0.607	−0.134	0.307	−0.833	−0.151	0.212
	R^2^ = 0.30	(P<0.001)		R^2^ = 0.19	(P = 0.001)		R^2^ = 0.32	(P<0.001)	
				**After the**	**rainy season**				
Model		Y_1_			Y_2_			Y_3_	
	Unstandardized coefficients B	Standardized coefficients (Beta)	Sig.	Unstandardized coefficients B	Standardized coefficients (Beta)	Sig.	Unstandardized coefficients B	Standardized coefficients (Beta)	Sig.
Constant	11.426		0.111	−5.811		0.452	−9.045		0.41
X_1_	−2.01	−0.178	0.041	−2.763	−0.221	0.01	−3.688	−0.206	0.015
X_2_	0.313	0.11	0.201	−0.132	−0.042	0.618	0.294	0.063	0.44
X_3_	6.30E–06	−0.034	0.692	1.80E–05	−0.089	0.285	1.60E–05	−0.054	0.508
X_4_	0.007	0.125	0.278	0.023	0.359	0.002	0.027	0.291	0.009
D_51_	−0.622	−0.088	0.307	−1.161	−0.149	0.08	−1.81	0.162	0.053
D_52_	−2.374	−0.2	0.021	−2.372	−0.181	0.032	−4.166	−0.222	0.008
D_53_	−2.599	−0.305	0.001	−4.807	−0.51	0	−6.862	−0.467	0
D_61_	−2.082	−0.506	0	−1.512	−0.332	0.004	−3.14	−0.476	0
	R^2^ = 0.37	(P<0.001)		R^2^ = 0.39	(P = 0.001)		R^2^ = 0.43	(P<0.001)	

Dependent Variable: Y_1_ (soil water content of 0–20 cm layer).

Y_2_ (average soil water content of 20–160 cm layer).

Y_3_ (average soil water content of 160–300 cm layer).

Independent Variables: X_1_ = Sin(gradient), X_2_ = Sin(aspect), X_3_ = flowaccu, X_4_ = elevation.

Dummy Variables: X_5_ = terrace, (D_51_, D_52_, D_53_) = (0,0,0); X_5_ = abandoned farmland, (D_51_, D_52_, D_53_) = (1,0,0).

X_5_ = grassland, (D_51_, D_52_, D_53_) = (0,1,0); X_5 = _woodland, (D_51_, D_52_, D_53_) = (0,0,1).

X_6_ = ridge, (D_61_) = 1; X_6_ = gully, (D_61_) = 0.

D represents sub-variable; binary variables 0 and 1 for the absence and presence of some land-use type or landform, respectively.

Before the rainy season, average SWC in the lower soil layer (10–20 cm) was significantly lower in grassland and woodland than in terrace, with no significant difference between abandoned farmland and terrace (*P*_D51_ = 0.109, *P*_D52_ = 0.003, *P*_D53_ = 0.047, *P*_X1_ = 0.013, and *P*_D61_ = 0.005; Table 5). Additionally, average SWC decreased with increasing gradient, with higher levels on the ridge than in the gully.

In the lower soil layer (20–160 cm), average SWC decreased significantly with increasing gradient (*P* = 0.026; Table 5), and was significantly lower in woodland than in the other three types of land-use types, with no significant differences among the latter three types. Other environmental factors had no significant linear correlation with average SWC (*P>*0.05).

In the deeper soil layer (160–300 cm), there also existed a significantly negative correlation between Sin(gradient) and average SWC (*P* = 0.001; Table 5). Average SWC obviously increased with increasing gradient and was significantly higher in terrace than in abandoned farmland, grassland, and woodland (in descending order).

Similar results can be seen in the data collected after the rainy season. That is, land-use type was the major environmental factor affecting average SWC, whereas landform and altitude strongly affected average SWC only in specific periods and soil layers (Table 5). In the whole vertical profile (0–300 cm), SWC occurred at high levels from upper to deeper layers in terrace, with the lowest level in woodland. Compared with data of terrace, average SWC was relatively low in grassland and woodland in the top (0–20 cm) and deeper soil layers (160–300 cm), with significantly low levels in abandoned farmland soils in the deeper layer only. In the deeper soil layer (160–300 cm), average SWC varied with different land-use types in descending order of terrace > abandoned farmland > grassland > woodland.

## Discussion

### 1 Vertical profiles of SWC at the catchment scale

According to Wang [21], the variability of SWC (as indicated by the CV) varies notably across the whole Loess Plateau, i.e., 15% in Changwu and 55% in Shenmu. In the small catchment of Sanyanjing, SWC profiles exhibited weak and medium degrees of variability at 0–300 cm depth [33], with CV in the range of 3–24% (Figure 2). The lower variability of SWC profiles in our study area may be related to the higher SWC levels across the catchment (Pearson correlation coefficient between average SWC and CV, −0.40; *P*<0.01). Qiu [17] found that wetter soil with greater vertical variations in an increasing trend along the SWC profile (mean 13.03%; and STD, 2.3%) is representative in a dry year in Danangou catchment on the Loess Plateau, where the land-use pattern (including slope farmland, terrace, and orchard) differs from that in our study area.

Cluster analysis of the mean and K values provides a clear description for the overall variability of SWC in the vertical profile. Based on combined grouping, the 101 vertical SWC profiles were classified into nine groups with high, medium, and low levels associated with increasing, stable, and decreasing trends (Table 3). More than half of the SWC profiles were obtained from terrace soils in the gully and classified into group 8 (58/101 before the rainy season and 80/101 after the rainy season) with high-level SWC in a stable trend (Tables 3, 4). Despite that all sampling points of woodland were also located in the gully, their average SWC remained the lowest among different land-use types and mostly descended along the vertical profile (Table 4). The above differences can be attributed to the lower soil water consumption by maize crop in the terrace, which generally has shallower root distribution and less above-ground biomass than trees in the woodland. Our observations coincide with previous findings on the Loess Plateau that soil water conditions of terrace, gully farmland, and dam land are better than that of artificial woodland. The latter land-use type is associated with soil desiccation, especially in deep soil layers [2], [22], [35], [36].

Although the cluster analysis divided vertical SWC profiles into nine groups, only eight types were present in the Sanyanjing catchment and no sampling points were classified into group 3 (i.e., low-level SWC with an increasing trend from top to bottom). According to previous research in semi-arid regions, if SWC occurs at low level in the upper soil layer, deep-root crops, shrubs, and trees will consume more soil water in the deeper soil layers through root extraction [37]–[40]. Additionally, it is hard to achieve soil water recharge in the deeper soil layers by precipitation infiltration because the depth of soil water infiltration is shallow. Therefore, soil desiccation exists in the lower soil layer in case of no groundwater recharge [8]–[10]. These mechanisms explain the absence of high-level SWC with an increasing trend along the vertical profile in the small catchment of Sanyanjing (Table 3).

### 2 Effects of environmental factors on average SWC at the catchment scale

Consistent with cluster analysis (Table 3), multiple regression analysis showed that land-use type had a significant effect on soil water status in the small catchment of Sanyanjing (Table 4, 5). This result coincides with the data previously reported in small catchments on the Loess Plateau [17], [18], [22], [23], [29]. For example, Zhang [23] concluded that average SWC (20–200 cm) descends with different land-use types (farmland > grassland > shrub land > and woodland, n = 80) in the small catchment of Zhifanggou. Bai [42] found that average SWC (0–500 cm) ranges from of 9% to 16% in orchard, gradient farmland, terrace, and grassland, but remains less than 10% in shrub land and most woodland (n = 91) in Nangou catchment in the central area of Loess Plateau, Ansai, Shaanxi. The consistency of the data demonstrates that cluster analysis is a reliable method for characterization of SWC profiles.

The effect of land-use type on SWC can be related to the differences existing in anthropogenic activity and vegetation type [22]. Average SWC was found significantly higher in terrace and abandoned farmland than in grassland and woodland along the 0–300 cm profile (Table 5). Abandoned farmland and terrace are associated with artificial tillage in the surface soil layer, which improves soil porosity and loosens soil structure, further enhancing soil water infiltration [22], [43]. Additionally, soil water consumption by crops is less than that in grassland and woodland due to lower leaf area index [21], contributing to the accumulation of SWC. The above mechanisms account for the greater average of SWC profiles with a stable trend to soil depth in terrace and abandoned farmland.

Difference in root distribution is another factor contributing the effect of land–use type on SWC [40]. In the Sanyanjing catchment, average SWC of woodland was higher in the 0–20 cm soil layer but lower in the 20–160 and 160–300 cm soil layers than data of grassland (Table 5). The varying trends of SWC profiles between grassland and woodland can be related to different distribution of root system in individual soil layers and stratified root extraction of soil water. The rooting depth of maize crop is reported to be approximately 100 cm and most maize roots are distributed in the soil layer of 0–20 cm, shallower than average rooting depths in grassland (20–60 cm) and woodland (20–100 cm) [40]. Diverse root distribution patterns can lead to different levels of soil water consumption by plants, contributing to great variability of SWC level.

In addition to land-use type, topographic factors strongly affected SWC in the study area (Table 4). This is because the distribution of wind and solar radiation varies with different topographic conditions, leading to different levels of soil evaporation, runoff on gradient, and soil water infiltration [41]. Gradient negatively affected SWC in the soil layers of 0–20, 20–160, and 160–300 cm (Table 5), possibly due to the increased runoff with increasing gradient and resultant reduction of precipitation infiltration [17], [27], [29], [41]. Other topographic factors including aspect and flow accumulation had no significant effects on average SWC in the three soil layers (Table 5). Similarly, Gómez [29] referred that aspect has no obvious influence on SWC in burned and unburned areas. Shi [19] suggested that aspect and catchment area significantly affect SWC during the wet period only, whereas elevation has a significant effect on SWC in arid and humid periods but not in semi-arid and semi-humid periods. In the present study, we found the effect of elevation on SWC of the three layers varying with the period of time and being significant after the rainy season only.

As for the landform type, location of sampling points significantly affected SWC only in the surface layer (0–20 cm) before the rainy season and throughout all the three layers (0–20, 20–160, and 160–300 cm) after the rainy season, with greater values in the gully than on the ridge (Table 5). The effect of landform type on SWC can be related to different levels of soil evaporation as affected by wind strength and solar radiation and soil physical properties. Similarly, Zhang [22] suggested that average SWC descends with different landforms as gully > terrace > slop land > hill top.

Overall, land-use type is the most significant factor affecting SWC while topographic factors and landform type are interacting jointly at the catchment-scale. Because the impact of environmental factors on SWC varies in different periods, it is necessary to increase the observation frequency, in order to better understand the spatiotemporal distribution and influencing factors of SWC in the small catchment. Such work will provide reference data for selecting reasonable environmental parameters in catchment scale SWC simulation over different periods of time.

## Conclusions

In this study, cluster analysis enables catchment-scale characterization of soil water profiles in terms of average level and variation trend along the vertical profile, allowing for simple and clear interpretation of the results. A total of nine groups of soil water profiles are recognized but those with low-level soil water content and a decreasing trend are not present in the Sanyanjing catchment. Land-use type, gradient, landform type, and altitude are the major environmental factors significantly influencing average soil water content in the hilly and gully catchment with complex terrain. The former two factors strongly affect soil water content along the 0–300 cm soil profile, whereas effects exerted by the latter two factors vary by soil layer and season.

Understanding the vertical profile of soil water content and evaluation of related major influencing factors in individual soil layers can help with sustainable land use and water management in catchment areas on the hilly and gully Loess Plateau as well as in arid and semi-arid areas with complex terrain. For better estimation of soil water profiles in small catchments, other factors such as fertilization, coverage, and soil physical properties may be considered with respect to specific soil layers.
